# Epigenetic inactivation and aberrant transcription of *CSMD1 *in squamous cell carcinoma cell lines

**DOI:** 10.1186/1475-2867-5-29

**Published:** 2005-09-09

**Authors:** Toni M Richter, Benton D Tong, Steven B Scholnick

**Affiliations:** 1 Dept of Otolaryngology - Head and Neck Surgery, Washington University School of Medicine, Box 8115, 660 S. Euclid Ave., St. Louis, MO 63110, USA

## Abstract

**Background:**

The p23.2 region of human chromosome 8 is frequently deleted in several types of epithelial cancer and those deletions appear to be associated with poor prognosis. *Cub and Sushi Multiple Domains 1 *(*CSMD1*) was positionally cloned as a candidate for the 8p23 suppressor but point mutations in this gene are rare relative to the frequency of allelic loss. In an effort to identify alternative mechanisms of inactivation, we have characterized *CSMD1 *expression and epigenetic modifications in head and neck squamous cell carcinoma cell lines.

**Results:**

Only one of the 20 cell lines examined appears to express a structurally normal *CSMD1 *transcript. The rest express transcripts which either lack internal exons, terminate abnormally or initiate at cryptic promoters. None of these truncated transcripts is predicted to encode a functional *CSMD1 *protein. Cell lines that express little or no *CSMD1 *RNA exhibit DNA methylation of a specific region of the CpG island surrounding *CSMD1*'s first exon.

**Conclusion:**

Correlating methylation patterns and expression suggests that it is modification of the genomic DNA preceding the first exon that is associated with gene silencing and that methylation of CpG dinucleotides further 3' does not contribute to inactivation of the gene. Taken together, the cell line data suggest that epigenetic silencing and aberrant splicing rather than point mutations may be contributing to the reduction in *CSMD1 *expression in squamous cancers. These mechanisms can now serve as a focus for further analysis of primary squamous cancers.

## Background

*CUB and Sushi Multiple Domains 1 *(*CSMD1*) was cloned as a candidate tumor suppressor or progression gene from a region of human chromosome 8 deleted in tumors of the upper aerodigestive tract, prostate, ovary and bladder [1-7]. Deletion of 8p23.2 or reduced expression of *CSMD1 *has been associated with poor prognosis in head and neck squamous cell carcinomas and in prostate cancers [2,5,8].

*CSMD1*, consisting of 70 exons spread over two megabases of 8p23.2, encodes a rare 11.5 kb transcript most abundantly expressed in the brain [1]. It is the founding member of a novel, evolutionarily highly conserved gene family whose proteins contain multiple domains thought to be sites of protein-protein or protein-ligand interactions and whose structure suggests that they may be transmembrane receptors or adhesion proteins [9,10].

Tumor suppressor genes are expected to be inactivated in cancers either genetically by mutations or epigenetically by modification of their promoters. While *CSMD1 *transcripts are detectable in upper aerodigestive tract epithelium, preliminary analysis of several head and neck squamous cell carcinoma cell lines suggested that *CSMD1 *expression was lost in these lines [1]. Although the region containing *CSMD1 *is frequently deleted in head and neck squamous cell carcinomas and prostatic adenocarcinomas [3,11-15], point mutations in the gene are relatively rare in primary squamous cancers [16] and in squamous cell carcinoma cell lines (Schmidt, Richter and Scholnick, unpublished). Nonsense or splice junction mutations in *CSMD1 *have not been reported and not enough is known about the function of the protein to accurately assess the effect of the few missense mutations that have been detected. Thus, if *CSMD1 *is inactivated in tumors, alternative mechanisms for gene silencing must be operating.

In this paper, we demonstrate that while most squamous cell carcinoma cell lines do not express full length *CSMD1 *transcripts, nearly all produce abnormal transcripts unlikely to encode functional CSMD1 proteins. Methylation of the DNA preceding *CSMD1*'s first exon is correlated with reduction in the level of expression and cell lines expressing at low levels do not appear to elongate the full 11.5 kb transcript. Other anomalies of expression include incorrect splicing and the use of cryptic promoters. Our data suggest that activation of these promoters may result from the global demethylation of the genome associated with tumorigenesis (reviewed by Ehrlich [17]).

Taken together these data demonstrate that mechanisms other than point mutation are responsible for the aberrant *CSMD1 *expression in head and neck squamous cell carcinoma cell lines, and these data suggest potential targets for further investigation in primary tumors.

## Results

### CSMD1 promoter methylation in HNSCC cell lines is correlated with expression levels

**Figure 1:**
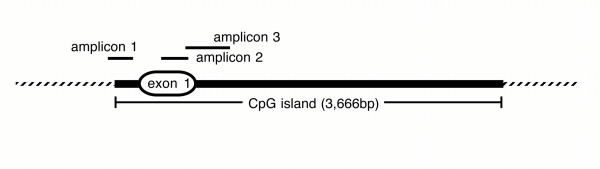
Positions of the amplicons used for COBRA and bisulfite sequencing relative to *CSMD1*'s first exon and the CpG island. Amplicon 1 extends from -395 to -112 bp, amplicon 2 from +175 to +396 bp, and amplicon 3 from +398 to +718 bp relative to the first base of the transcript. The region between amplicons 1 and 2 is so GC rich that no workable PCR primers could be designed to amplify it after bisulfite conversion. Transcription of *CSMD1 *is from left to right in the figure.

Preliminary evidence suggested that *CSMD1 *expression is lost in head and neck squamous cell carcinomas [1] but that point mutations were rare [[16], and Schmidt, Richter and Scholnick, unpublished]. To date, only two of the 20 cell lines we have tested for *CSMD1 *expression, UPCI:SCC066 and PCI-13, express large transcripts initiated at the normal *CSMD1 *promoter. These data suggest that a mechanism(s) other than point mutation must be responsible for the loss of expression. *CSMD1*'s first exon is embedded in a 3.7 kb CpG island (data from the UCSC genome browser [18]) suggesting that promoter methylation might epigenetically silence the gene. To test this hypothesis, we surveyed 32 head and neck cancer cell lines for *CSMD1 *promoter methylation using the Combined Bisulfite Restriction Analysis (COBRA) technique described by Xiong and Laird (Methods) [19]. COBRA analysis of the three amplicons diagrammed in Figure 1 suggested that 28 of the cell lines (87%) had more promoter methylation than did normal upper aerodigestive epithelium (data not shown).

We selected nine of these cell lines for high resolution analysis of promoter methylation by sequencing of clones from bisulfite converted genomic DNA. This approach has the distinct advantage of allowing determination of the state of all the CpG dinucleotides within an amplicon on an allele by allele basis. Amplicons 1 and 2 have 19 and 20 CpG dinucleotides, respectively. Amplicon 3 could not be examined by this technique because it is unclonable after bisulfite conversion. The methylation data were correlated to *CSMD1 *expression levels as measured by quantitative RT-PCR using an amplicon spanning exons 1 and 2 (Methods). A pool of cDNA from five normal oropharyngeal epithelium specimens served as a basis for comparison to the cell lines.

**Figure 2:**
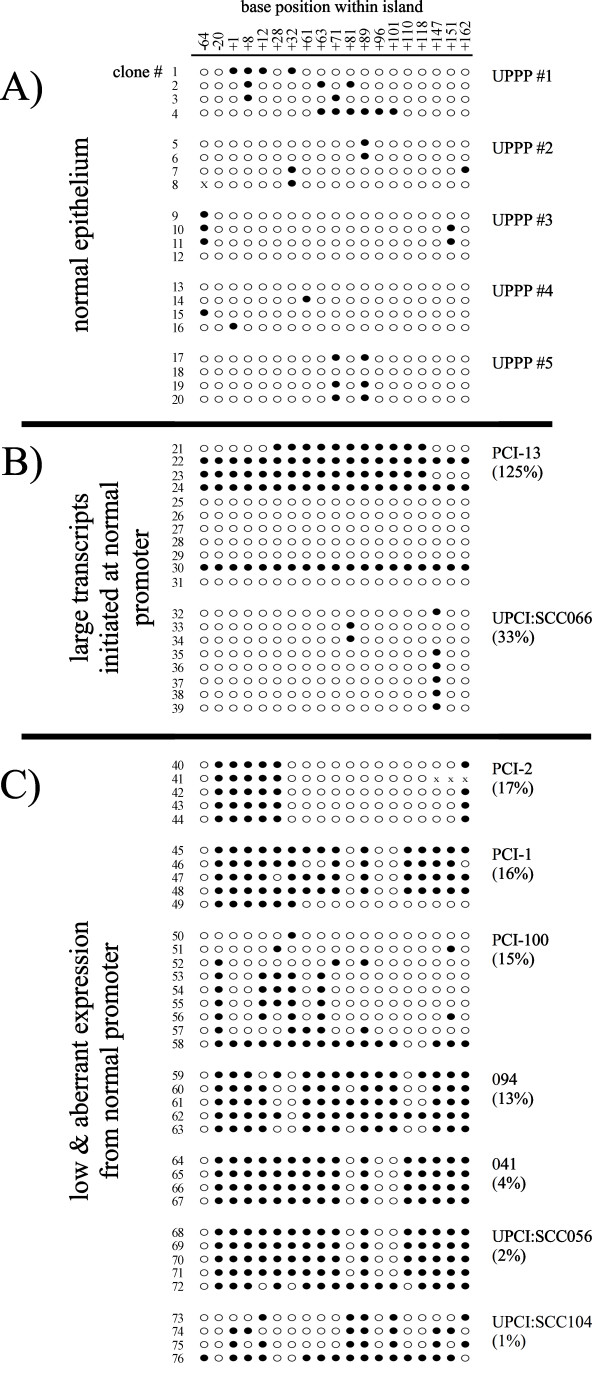
Methylation status of each of the 19 CpG dinucleotides in amplicon 1. Each CpG dinucleotide within the amplicon is represented by a column and its position in base pairs relative to the CpG island is given at the top of the figure. Positions were measured from the end of the CpG nearest the 5' end of CSMD1 as defined by data obtained from the UC Santa Cruz genome browser (island sequence beginning CGGTGTGCGGCGTGAGCTTCCCCCACCCGAG...). Each sequenced clone is represented by a row and is numbered sequentially starting from the top of the figure. Open symbols (○) indicate that cytosine residue of the dinucleotide was unmethylated, closed symbols (●) indicate that it was methylated, and "x" indicates that the identity of the base could not be determined from that sequencing reaction. The level of expression is presented as a percentage relative to five pooled samples of normal UPPP epithelium. These are not the same UPPP samples used for measurement of promoter methylation in normal tissue. A) normal oropharyngeal epithelium from five individuals undergoing uvulopalatopharyngoplasty. B) Methylation status of two cell lines that express large *CSMD1 *transcripts from the normal promoter (PCI-13 and UPCI:SCC066), C) cell lines with low expression of the *CSMD1 *transcript from the normal promoter (PCI-2, PCI-1, PCI-100, 094, 041, UPCI:SCC056, and UPCI:SCC104).

Our data from amplicon 1 demonstrate a clear relationship between methylation and the level of expression (Figure 2). The bisulfite sequencing data confirm that there is relatively little promoter methylation in normal tissue (clones 1–20, Figure 2A). This is also the case in cell line UPCI:SCC066 which expresses a large *CSMD1 *transcript from the normal promoter at a level approximately 33% of that of normal tissue (clones 32–39, Figure 2B). PCI-13, our highest expressing line at 125% of normal epithelium, displays two distinct patterns of promoter methylation with some clones heavily methylated (clones 21–24, and 30) and others with no methylation (clones 25–39 and 31; Figure 2B). This pattern is consistent with either heterozygosity for methylation or the co-existence of 2 distinct populations within the cell line, one heavily methylated and one unmethylated. We cannot distinguish between these two possibilities using the currently available data.

The remaining cell lines express *CSMD1 *at a level half that of UPCI:SCC066 or less (ranging from 17% to 1% of normal epithelium) and they exhibit considerably greater methylation of amplicon 1 (clones 40–76, Figure 2C). Cell lines with more amplicon 1 methylation tend to express the gene at lower levels but the relationship is not strictly quantitative (Figure 2C).

In contrast, our data revealed no relationship between expression level and methylation of amplicon 2 (located towards the 3' end of exon 1, Figure 1). For example, all of the 10 clones of amplicon 2 sequenced from PCI-13 were methylated at 19 or 20 of their 20 CpG dinucleotides. UPCI:SCC066, on the other hand, has nearly no methylation in amplicon 2 with only a single methylated CpG dinucleotide detected in one clone out of the nine sequenced. Amplicon 2 ranges from completely unmethylated to heavily methylated in the seven remaining cell lines (data not shown).

### Low transcript levels are accompanied by a failure to elongate the full CSMD1 transcript

On the surface, the quantitative RT-PCR data presented in Figure 2 suggest that the cell lines we consider low expressing might still have up to 17% of the normal level of *CSMD1 *transcript. A survey of 20 cell lines using a battery of RT-PCR primer pairs located throughout the 11.5 kb transcript reveals that this is not the case. These lines included OKF6-TERT1, a TERT immortalized, p16 deficient but untransformed oral keratinocyte cell line [20].

Our data suggest that the low expressing cell lines shown in Figure 2C express considerably more of the 5' end of the 11.5 kb transcript than they do exons further 3', a phenomenon well illustrated by cell line PCI-100. This line expresses the exon 1/exon 2 amplicon at approximately 15% of the level of normal epithelium. In contrast, we had previously reported that *CSMD1 *transcripts were not detectable in this line by combined RT-PCR and Southern blotting using three sets of intron spanning primers [1]. The most 5' of those amplicons spans exons 9 through 26.

**Figure 3:**
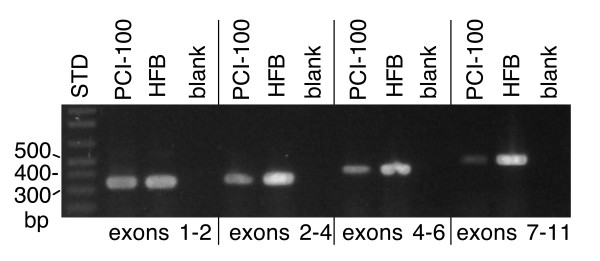
RT-PCR data demonstrating the preferential loss of longer *CSMD1 *transcripts in cell line PCI-100. Exons 1 and 2 were amplified with prm1542 (gggacccgatgctatgagagggaag) and prm1482 (cccgtgaggaaaccctgggctct), exons 2 through 4 with prm2078 (gggcgagcgcaataggatacagtt) and prm1382 (ggatggcgtggccttccaagatgtag), exons 4 through 6 with prm1392 (agctgcctccctggctacatcttgg) and prm1405 (cttggaactgagcgttaaatcctttg), and exons 7 through 11 with prm1463 (tgaaaaaggcgattgagttgaagtc) and prm1421 (gaccgatctggtgtctcccaccttc). "STD" indicates a lane of DNA standards whose sizes are given on the left side of the figure, "HFB" = human fetal brain, "blank" indicates a control reaction with water substitute for cDNA.

Analysis with additional primer pairs resolves this apparent paradox by demonstrating that the amount of transcript declines sharply and reproducibly as one examines progressively more 3' exons (Figure 3). The same effect is seen using either oligo-dT or random hexamer primed cDNA. No transcript of this structure has been detected in normal epithelium nor have we detected any sequence alterations in PCI-100 that would explain why the full transcript is not expressed. It is not clear whether PCI-100 produces a small number of discrete size classes of transcript, if individual transcripts terminate at random points within the very large introns in this part of the gene (the first 10 introns average ~150 kb in length), or if the short transcripts result from the elevated activity of a previously undetected posttranscriptional control mechanism.

### Inactivation of CSMD1 by aberrant splicing

**Figure 4:**
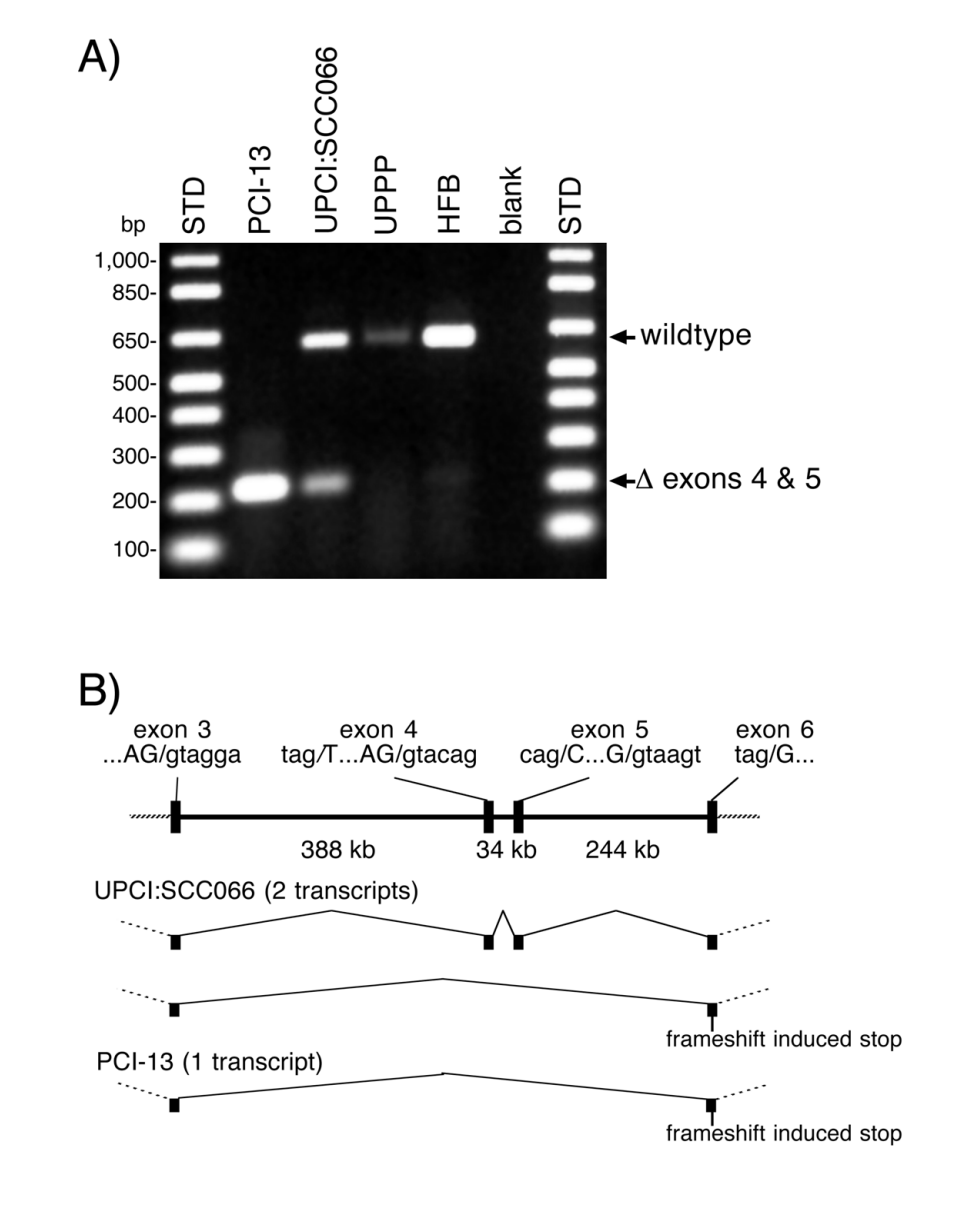
A) RT-PCR demonstrating deletion of exons 4 and 5 from the *CSMD1 *transcripts of cell lines PCI-13 and UPCI:SCC066. RT-PCR with a forward primer in exon 3 (prm2080, gggatttcagctgccctcctctat) and a reverse primer in exon 6 (prm1405, cttggaactgagcgttaaatcctttg) yields a 623 bp product from upper aerodigestive tract (UPPP), human fetal brain (HFB) and UPCI:SCC066 RNA. Exclusion of exons 4 and 5 from the transcripts of PCI-13 and UPCI:SCC066 reduces the size of the PCR product to 220 bp. The sizes of the DNA standards (STD) are indicated on the left side of the figure. The blank reaction contained water instead of cDNA. "UPPP" = normal oropharyngeal epithelium isolated from uvulopalatopharyngoplasty surgical discards. B) Idiogram showing the organization of exons 3 through 6 in the human genome. The sizes of the introns were obtained from the UCSC genome browser. The sequences of the splice junctions are provided above the idiogram with intronic bases in lowercase and exonic bases in uppercase. The structure of the UPCI:SCC066 and PCI-13 *CSMD1 *transcripts are shown below the idiogram.

Two cell lines, UPCI:SCC066 and PCI-13, express large *CSMD1 *transcripts initiated at the normal promoter. Subsequent finer scale analysis demonstrates that PCI-13's transcript lacks exons 4 and 5, resulting in a frameshift-induced nonsense codon in exon 6 (Figure 4). Sequencing of the PCI-13 RT-PCR product demonstrates the direct juxtaposition of wildtype exons 3 and 6 and that the transcript contains no novel sequences or splices that would prevent the frameshift. UPCI:SCC066 produces two transcripts, a normal one that includes exons 4 and 5 and another that lacks them (Figure 4). RT-PCR of human fetal brain cDNA reveals very low levels of an RT-PCR product corresponding in size to that expected from the internally deleted transcript. This band is not readily visible at the exposure used for Figure 4A. We have not detected a similar sized PCR product in RNA from oropharyngeal epithelium but this may reflect the fact that *CSMD1 *transcripts are ~10x less abundant in oropharyngeal epithelium than they are in fetal brain (data not shown).

The transcripts lacking exons 4 and 5 appear to result from aberrant splicing rather than somatic deletion of these two exons or mutations of their splicing consensus sequences. Exons 4 and 5 can be amplified from PCI-13 genomic DNA and sequencing of those PCR products demonstrates that both their coding sequences and consensus splice sites are wildtype.

### Activation of cryptic promoters in cancer cell lines

**Figure 5:**
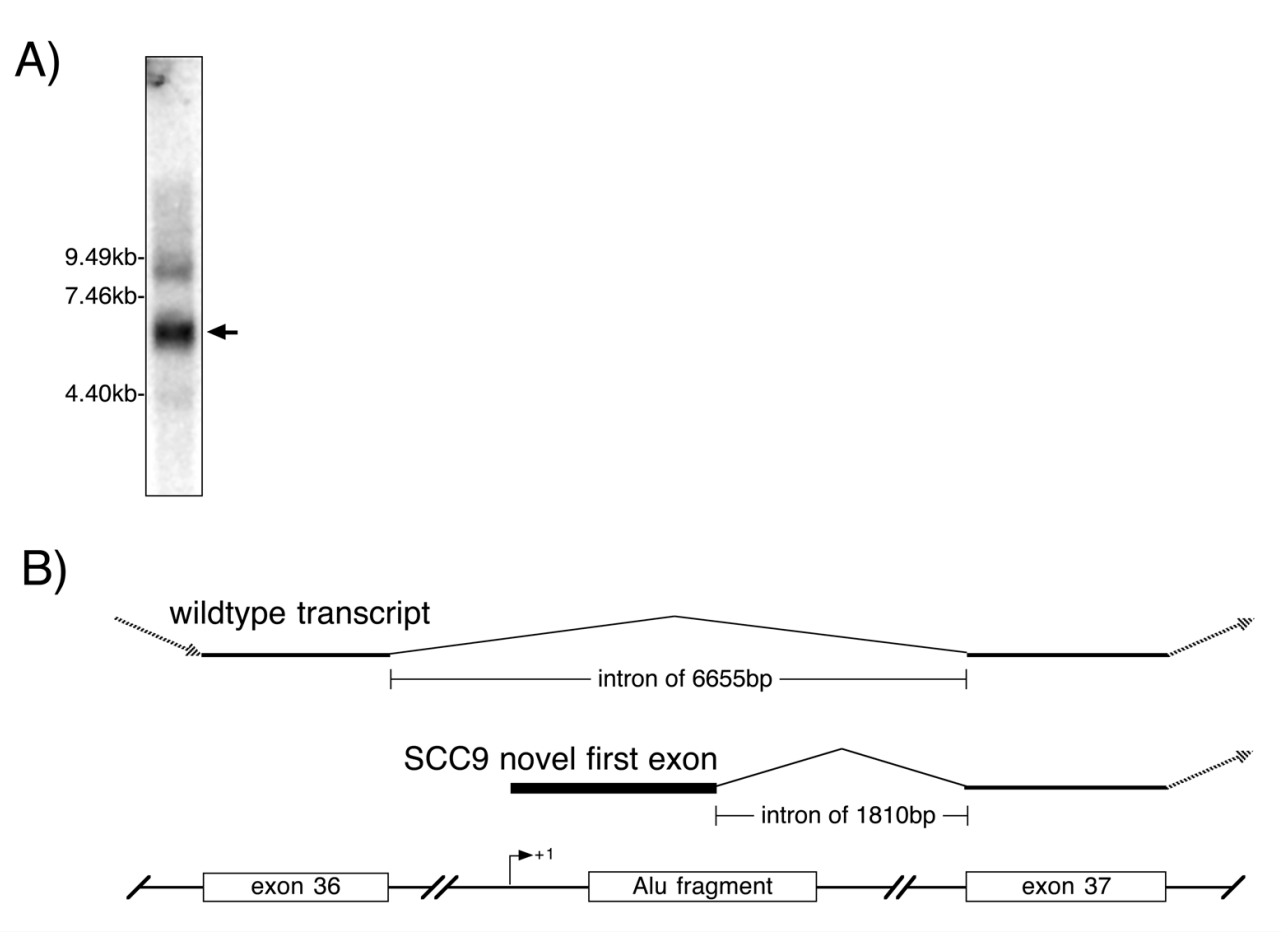
A) Northern blot showing the truncated *CSMD1 *transcript expressed by the SCC9 cell line. Approximately 8 μg of poly-A+ RNA was fractionated on a denaturing 1% agarose gel, transferred to a filter and probed with the full length human *CSMD1 *cDNA. The image was acquired by phosphorimager scanning. B) Structure of the aberrant SCC9 transcript and the corresponding segment of the wildtype *CSMD1 *transcript showing the relationship between the novel cryptic promoter and exon 37. The partial Alu element contains only the first 120 base pairs of the normal ~300 bp Alu sequence.

The RT-PCR survey revealed a second transcriptional anomaly exhibited by 4 cell lines: SCC9, 041, PCI-1 and PCI-2. Like PCI-100, these lines express low levels of the very 5' end of the transcript and even lower levels of more 3' exons within the first half of the transcript. However, these lines are distinct in expressing higher levels of the 3' half of the transcript, suggesting that alternative promoters in the middle of the gene may be used. SCC9 was chosen for further study because it expresses the 3' half of the transcript at a level dramatically higher than normal for oropharyngeal epithelium. Northern blotting detects a comparatively abundant 6.4 kb truncated transcript as well as smaller amounts of an 8.7 kb transcript in SCC9 (Figure 5A). The other three cell lines express their truncated *CSMD1 *transcripts at lower levels (data not shown). Sequence analysis of *CSMD1 *cDNA clones from SCC9 demonstrates that many transcripts are improperly spliced, resulting in retention of intronic sequences and/or deletion of exonic sequences (data not shown). In particular, retention of sequences from intron 40 is common. The high frequency of faulty splicing may explain the broadness of the 6.4 kb *CSMD1 *band in Figure 5A and suggests that the 8.7 kb transcript may also be incompletely or improperly spliced.

5' RACE [21] reveals that the SCC9 message is initiated just upstream of an Alu element in intron 36 (Figure 5B). Only the 5'-most 120 base pairs of the Alu element are present in the genome. RT-PCR using a forward primer specific for the novel sequences of the SCC9 transcript (prm1904, cgtttagttcgacacacttcatgt) demonstrates that cell lines 041, PCI-1 and PCI-2 do not initiate their *CSMD1 *transcripts at the same point, suggesting that other cryptic promoters are active in these lines. The sequence of this novel exon has been entered in Genbank as accession number DQ093422.

### DNA methyltransferase inhibitors activate the same cryptic promoter used in cell line SCC9

Expression from epigenetically silenced promoters can sometimes be restored by treatment with inhibitors of DNA methyltransferase or histone deacetylase activity ([22]). We selected two low expressing cell lines with promoter methylation, UPCI:SCC104 and 094, for treatment with various concentrations of 5-azacytidine or 5-aza-2'-deoxycytidine (5-aza-dC) as well as combinations of either of those drugs with the histone deacetylase inhibitor trichostatin A. These treatments did not reactivate the silenced *CSMD1 *promoter. COBRA analysis of genomic DNA from the treated cells suggested that the drugs did not robustly affect methylation of the *CSMD1 *promoter even at levels high enough to be toxic to the cells. These experiments did however shed light on the cryptic promoter used in SCC9 cells and on the interpretation of experiments using methyltransferase inhibitors.

**Figure 6:**
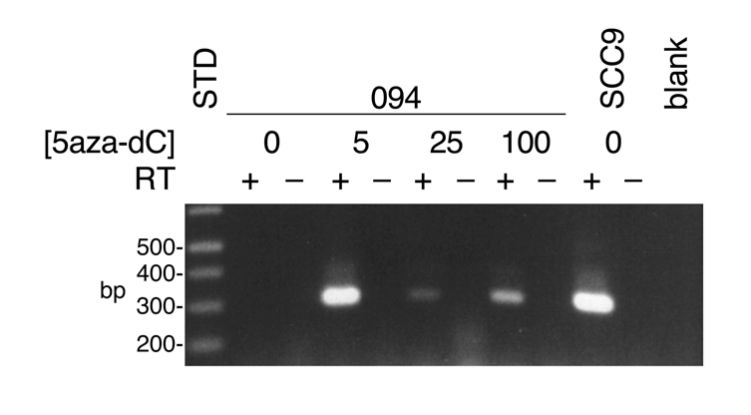
5aza-dC induced activation of *CSMD1 *from the same cryptic promoter active in cell line SCC9. Cultures of cell line 094 were treated with the indicated micromolar concentrations of 5aza-dC, and used for RNA extraction. Cell line SCC9 was grown in the absence of the drug and used as a control. RT-PCR was performed with primers prm1904 (cgtttagttcgacacacttcatgt) and prm1880 (cactggaaggagagcacgtcgttcac). prm1904 is specific for the cryptic first exon first used in SCC9, prm1880 is homologous to *CSMD1 *exon 38. The presence or absence of reverse transcriptase in the RT reaction is indicated by a + or -, respectively. The blank reaction contained water instead of input cDNA. The sizes of the markers are indicated on the left side of the figure.

Treatment of cell line 094 with relatively high doses of 5aza-dC results in the expression of the 3' end of the *CSMD1 *transcript. This transcript was not detected in control 094 cells undergoing mock drug treatment (Figure 6) nor was it detected in 094 cells growing under normal culture conditions (data not shown). RT-PCR of cDNA from drug-treated 094 cells using the primer developed from the novel 5' exon of SCC9's truncated transcript (prm1904, see above) yielded a product identical in size to that amplified from SCC9 cDNA (Figure 6). The identity of the product was confirmed by DNA sequencing which also revealed that the drug-induced 094 transcript was more faithfully spliced than the transcript expressed in SCC9 (data not shown).

## Discussion

Our data clearly demonstrate that expression of normal *CSMD1 *transcripts is rare in head and neck squamous cell carcinoma cell lines. Of the HNSCC cell lines examined, only UPCI:SCC066 appears to express a normal transcript from the expected promoter. Even that cell line produces a second species of aberrantly spliced transcript lacking internal exons. Our data suggest that epigenetic modification of the DNA 5' of the transcription start site may contribute to the down-regulation of *CSMD1*. In addition, a low level of expression appears to be associated with production of prematurely terminated transcripts. This degree of complexity might be expected from a 2 megabase, 70 exon gene.

Methylation of a specific region of the CpG island, -395 to -112 bp relative to the transcriptional start site (amplicon 1), appears to be correlated with the activity of the normal *CSMD1 *promoter. In contrast, methylation of amplicon 2, located within the first exon, shows no such relationship. Our data suggest that the relationship between the amount of methylation in amplicon 1 and the level of expression may not be strictly quantitative. Differences between cell lines with amplicon 1 methylation could arise through a number of mechanisms, for example, variations in the levels of transcription factors between cell lines. In cases where there is considerable heterogeneity in the methylation pattern within a cell line like PCI-100, alleles with less methylation may be expressed at higher levels than those more heavily methylated (compare clones 51 and 52 to clone 58 in Figure 2C). Alternatively, the presence of methylation in amplicon 1 could be a qualitative but not strictly quantitative indicator of methylation of a critical segment of the promoter not discovered in this study.

The normal *CSMD1 *promoter was not reactivated by drugs that inhibit DNA methyltransferases and histone deacetylases, nor did the drugs abolish *CSMD1 *promoter methylation, even at toxic doses. Not all genes with promoter methylation respond to such treatments [23]. These drug treatments did, however, provide a potential explanation for the use of a normally cryptic promoter by cell line SCC9. The *CSMD1 *transcript in this line is initiated near a partial Alu element. 5-aza-dC treatment of cell line 094 activates the same cryptic promoter. This suggests that cryptic promoters may be naturally activated by general hypomethylation of the genome in cancer cells and the subsequent release of repetitive elements from epigenetic repression (reviewed by Ehrlich [17]). The SCC9 transcript does not appear to encode a functional protein but, with a very large gene like *CSMD1*, there is a potential for some abnormally initiated transcripts to encode truncated proteins with dominant negative properties.

The second ramification of this finding is for the interpretation of data obtained by treating cells with methyltransferase inhibitors. Detection of *CSMD1 *transcripts solely with primers mapping to the 3' end of the gene could have been erroneously interpreted as representing reactivation of the normal promoter. It seems imperative that such experiments demonstrate that transcripts detected after drug treatment are actually initiated at the normal promoter.

Aberrant splicing also appears to play a role in the production of defective *CSMD1 *transcripts. Loss of splicing fidelity has been proposed as a characteristic of cancer cells [24,25] and this would be consistent with the variety of misspliced transcripts we detected from SCC9. However, the removal of exons 4 and 5 from the *CSMD1 *transcript in PCI-13 may reflect a more specific phenomenon than a general inability to splice large introns; this line is still capable of splicing large introns as evidenced by its successful splicing of exon 3 to exon 6, eliminating an intron of over 666 kb. The failure to include exons 4 and 5 may be due to inactivation of a splicing enhancer in intron 3, or to less efficient splicing due to the fact that exons 4 and 5 do not begin with the consensus G residue (Figure 4B) [26].

## Conclusion

Taken together, our data suggest that *CSMD1 *function is lost in head and neck squamous cell carcinoma cell lines through a variety of mechanisms other than point mutagenesis. Epigenetic modifications of amplicon 1 and defective splicing appear to be fruitful areas to explore in primary head and neck squamous cancers.

## Methods

### Cell lines and tissue samples

DNA from HNSCC cell lines UMSCC9, UMSCC35, UMSCC37, UMSCC38, UMSCC45, UMSCC49, UMSCC65, UMSCC68, and UMSCC76 was provided by Dr. Thomas Carey, University of Michigan [27]. Dr. Ruud Brakenhoff, Vrije Universitat, provided cell lines 040, 041, and 094 [28]; Dr. Theresa Whiteside, University of Pittsburgh, provided cell lines PCI-1, PCI-2, PCI-4B, PCI-13, PCI-30, PCI-50, PCI-51, PCI-52, PCI-100 [29], SCC4, SCC9 [30,31], and UPCI:SCC068, UPCI:SCC74, UPCI:SCC104, UPCI:SCC182, UPCI:SCC203, and UPCI:SCC220 [developed by Dr. Susanne Gollin, University of Pittsburgh, 32]. Dr. Gollin provided cell lines UPCI:SCC056, UPCI:SCC066, and UPCI:SCC114 [14,16,32]. The immortal but untransformed keratinocyte line OKF6-TERT1 was obtained from Dr. James Rheinwald, Harvard University [20].

Normal oropharyngeal epithelium was isolated from discarded tissue from uvulopalatopharyngoplasties (UPPP) collected anonymously with the approval of the Washington University Human Studies Committee.

### Cell Culture and Tissue Preparation

Squamous cell carcinoma cell lines were grown in DMEM or DMEM:F-12, 1:1 Mixture (BioWhittaker) containing 10% fetal bovine serum (Sigma). DMEM medium was supplemented with 1X MEM Nonessential Amino Acids (BioWhittaker). Upper aerodigestive tract epithelium was separated from the rest of the UPPP specimen by digestion with Dispase II (Roche) using a protocol adapted from Oda and Watson [33].

### Nucleic acid preparation and bisulfite conversion

Genomic DNA was isolated by using either Nucleospin Tissue kits (Clontech), QIAamp DNA Blood Mini kits (Qiagen) or Trizol (Invitrogen) according to the manufacturers' instructions. Total RNA isolation, synthesis of first strand cDNA, RT-PCR and 5' RACE PCR were performed essentially as previously described [1]. Poly-A+ RNA for Northern blotting was selected from total RNA using Oligotex beads (Qiagen). Northern blotting and hybridization were performed as previously described [1]. cDNA synthesis was primed using oligo dT or random primers and extended by either Thermoscript or Superscript III reverse transcriptase (Invitrogen). PCR was run in Perkin-Elmer 480 or Applied Biosystems 9700 thermal cyclers for 35 cycles unless otherwise noted. Images of ethidium bromide stained gels were captured with a Gel-Doc imaging station (Biorad). Quantitative PCR was run in an Applied Biosystems 5700 thermal cycler using SYBR Green Master Mix (Applied Biosystems). Primers prm2426 (gtgtggagtatctgcagacatga) and prm2427 (ctggactaagcctccacagttct) were used to amplify a 132 base segment spanning *CSMD1*'s first and second exons. An amplicon from human 18S RNA was used as a basis for comparisons across cell lines (primers prm2396, ttcggaactgaggccatgat and prm2397, tttcgctctggtccgtcttg). Calculations were performed using the ΔΔCt method in GeneAmp 5700 SDS software (version 1.3) and Microsoft Excel. Quantitation was based on the average values obtained from duplicate reactions. The level of *CSMD1 *expression in normal oropharyngeal epithelium was determined from pooled cDNA from five UPPP specimens.

We used the CpGenome DNA Modification kit (Intergen) for bisulfite conversion of the genomic DNA according to the manufacturer's protocol, with the following exception. Incubation of the conversion reaction was carried out in a thermal cycler for six cycles each consisting of three minutes at 94°C followed by three hours at 50°C (Christina Menke and Paul Goodfellow, personal communication).

### Analysis of CSMD1 Promoter Methylation

Methylation of three segments of the *CSMD1 *CpG island was examined using the Combined Bisulfite Restriction Analysis technique (COBRA) [19]. All three segments were amplified by using the nested primers and PCR conditions listed in Table 1. Amplicon 1 extends from -395 to -112 bp, amplicon 2 from +175 to +396 bp, and amplicon 3 from +398 to +718 bp relative to the first base of the transcript. A small region surrounding the transcription start site (-111 to +174 bp) could not be examined because no PCR primers could be designed from its extremely GC-rich sequence. The first round PCR used 2 μl of bisulfite converted genomic DNA in a final volume of 10 μl. Subsequent amplifications with nested primers used 4 μl of first round product as template in reactions with a final volume of 20 μl. All PCR was carried out for 35 cycles. A portion of the second round PCR product was run on a 1.5% agarose gel, stained with ethidium bromide, and quantified using the ImageQuant software package (v1.2 for Macintosh, Molecular Dynamics) so that equal amounts of each could be used in restriction digests.

**Table 1:** PCR primers used for COBRA analysis of *CSMD1 *CpG island

PCR round	forward		reverse		annealing temp	[MgCl_2_] (mM)	amplicon size (bp)	# of CpG's	# *BstU1 *sites	# *Taq1 *sites
Amplicon 1										
1 st	prm1998	taagttaggtagggggttgtttt	prm1999	aaccactacaaaactaaactact	45°C	1.5	626			
2 nd	prm2000	ggaagggagattaaaggatgg	prm2001	aaactcaaccatccttacccacaa	58°C	1.5	298	19	1	4
Amplicons 2 and 3										
1 st	prm1942	gagtagtttagttttgtagtggt	prm1943	tattaaattcctttctccttaaca	45°C	2.0	594			
2 nd	prm2006	agtagtttagttttgtagtggtt	prm2007	caatcatatctacaaatactcc	45°C	2.0	225	20	2	1
2 nd	prm1954	tgtggagtatttgtagatatgattg	prm1968	cctttctccttaacaccctatacta	55°C	1.5	388	31	4	1

Restriction digests for COBRA were performed with either *Bst*U I or *Taq*^α ^I (5 or 10 units per reaction, respectively; New England Biolabs) for 4 hours in a final volume of 10 μl. *Taq*^α ^I digests were performed only when no methylation was detected with *Bst*U I. *Bst*U I digests also included an internal control DNA fragment to confirm complete digestion. This DNA fragment contains a single *Bst*U I site and was amplified from a cloned *CSMD1 *cDNA using primers prm2020 (agatcccccagtgtctccctgtgt) and prm2021 (actgctggtgccgtggtaatgact). The control PCR product is 1019 bp long and is digested to two fragments of 605 and 414 bp by *Bst*U I. Digestion products were fractionated on a 10% polyacrylamide gel, stained with ethidium bromide, and visualized with a Gel-Doc video imaging workstation (Bio-Rad).

High resolution analysis of methylation was performed by sequence analysis of individual clones from amplicons 1 and 2. DNA from amplicon 3 proved unclonable and gel electrophoresis suggests that its very AT rich sequence results in a bent DNA configuration. PCR products were purified using the Nucleospin Extraction columns (Clontech) and inserted into the pCR2.1-TOPO vector using the TOPO TA Cloning kit (Invitrogen) according to the manufacturer's instructions. Plasmid DNA from individual colonies was isolated using the Nucleospin Plus Plasmid Miniprep kit (Clontech) and sequenced with a reverse vector primer (agcggataacaatttcacacagga) using fluorescence based sequencing with Big Dye Terminator mix (Applied Biosystems).

### Treatment of cultured cells with DNA methyltransferase inhibitors

Cell line 094 was treated with 5-aza-2'-deoxycytidine (5aza-dC) (Sigma) dissolved in DMSO. Two 100 mm cell culture dishes containing 5 × 10^5 ^cells were established for each of the drug concentrations tested. Cells were grown for 72 hours in media containing DMEM:F-12, 1:1 Mixture (BioWhittaker) with 1X MEM Nonessential Amino Acids (BioWhittaker) and 10% fetal bovine serum and then switched to media containing 5aza-dC at concentrations of 0 μm, 5 μM, 25 μM, or 100 μM. Cells were fed daily for 4–5 days and then both plates were harvested in 3 ml of Trizol (Invitrogen) for isolation of RNA and DNA according to the manufacturer's instructions. RT-PCR used for detection of *CSMD1 *transcripts in these treated cells was run for 40 cycles.

## Abbreviations

5aza-dC = 5-aza-2'deoxycytidine, COBRA = Combined Bisulfite Restriction Analysis, *CSMD1 *= *Cub and sushi multiple domains 1*, HNSCC = head & neck squamous cell carcinoma, RT-PCR = reverse transcription – polymerase chain reaction, SCC = squamous cell carcinoma, UPPP = uvulopalatopharyngoplasty.

## Competing interests

The author(s) declare that they have no competing interests

## Authors' contributions

TMR performed the DNA methylation analysis, and parts of the transcript survey. BDT cloned and characterized the novel first exon expressed in cell line SCC9. SBS performed parts of the transcript survey and characterized the deletion of exons 4 and 5 in PCI-13. All three authors participated in the analysis of the data and in the writing of the manuscript.
